# Implant Placement after Closure of Oroantral Communication by Sinus Bone Graft Using a Collagen Barrier Membrane in the Shape of a Pouch: A Case Report and Review of the Literature

**DOI:** 10.3390/medicina57060626

**Published:** 2021-06-16

**Authors:** Jae-Ha Baek, Byung-Ock Kim, Won-Pyo Lee

**Affiliations:** Department of Periodontology, School of Dentistry, Chosun University, Gwangju 61452, Korea; qorwogk5@naver.com (J.-H.B.); bobkim@chosun.ac.kr (B.-O.K.)

**Keywords:** closure, fistula, maxillary sinus membrane, oroantral communication

## Abstract

Oro-antral communication (OAC) acts as a pathway for bacteria between the maxillary sinus and oral cavity, and is a common complication after the removal of a dental implant or extraction of a tooth from the maxillary posterior area. In the case of an untreated OAC, oro-antral fistula develops and becomes epithelialized. We aimed to introduce a treatment for OAC closure via a sinus bone grafting procedure using bone tacks and a collagen membrane with an allograft. The procedure was performed by applying an absorbable membrane made in pouch form. This membrane acted as a barrier for closing the large sinus membrane perforation. Bone tacks were used to fix the membranes. Subsequently, the maxillary sinus was filled with the allograft, and the absorbable membrane was reapplied. Primary closure was achieved by performing a periosteum-releasing incision for a tension-free suture. After 6 months, sufficient bone dimensions were gained without any occurrence of maxillary sinusitis or recurrence of OAC. Additional bone grafts and implantation could be performed to rehabilitate the maxillary posterior area. We conclude that this technique might be a useful treatment for reconstructing the maxillary posterior area with simultaneous sinus bone graft and OAC closure.

## 1. Introduction

Implant removal or extraction of a tooth from the maxillary posterior region leads to maxillary sinus perforation and oro-antral communication (OAC). OAC indicates a pathological condition in which the maxillary sinus and oral cavity are connected. This acts as a pathological path for bacteria and can cause maxillary sinusitis. If this condition persists, it progresses to epithelial patency, known as an oroantral fistula (OAF) [1,2]. If OAC and OAF remain unclosed, the oral bacterial infection persists and the continuous communication of food and foreign substances occurs, invoking chronic maxillary sinusitis without healing of the maxillary sinus membrane. As a result, there is a delay in maxillary posterior bone reconstruction for implant placement, and eventually a prolongation of the patient’s treatment period [3,4].

To date, various methods have been introduced for OAC and OAF treatments. In general, when the diameter of the path is less than 5 mm, spontaneous closure occurs. However, in cases wherein it is 5 mm or more, closure through a surgical intervention is required. A typical surgical procedure involves closure using a soft tissue flap [5,6], including the buccal advancement flap as reported by Rehrmann [7], the buccal fat pad (BFP) introduced by Egyedi [8], and the palatal pedicle flap designed by Ashley [9]. The risk of recurrence exists when using a single flap; hence, a double-layer closure procedure using more than one flap has also been introduced and reported in several previous studies. However, the use of a soft tissue flap involves lowering the depth of the buccal vestibule, and an insufficient amount of attached tissue. If future implant placement is scheduled, an additional surgery, such as ridge reconstruction, is required. Consequently, the treatment period for the patient is prolonged, and trauma inflicted on the surgical site increases [10].

Therefore, in this study, we introduced a new treatment modality to a patient affected by OAC in the maxillary posterior region. We aimed to introduce multiple implantations after effective OAC treatment by applying an absorbable membrane in the shape of a pouch followed by allograft use.

## 2. Case Description

A 36-year-old woman visited the hospital with the complaint of having pus and gingival swelling in #17. She had a history of pericarditis and long-term steroid use. After the administration of prophylactic antibiotics, #17 was extracted, following which the communication of the maxillary sinus was observed at the apex of the tooth. Thereafter, a collagen plug was inserted, and the defect was sutured with black silk. However, after approximately 2 months, the patient presented with an OAF in the form of a pinpoint, while the large size of the OAC remained. Instead of using soft tissue flaps for the OAC closure, the latter was planned simultaneously during the maxillary sinus bone graft by applying an absorbable membrane in the shape of a pouch, because the maxillary sinus was in a healthy state without any maxillary sinusitis, except for the OAC. We thus carried out guided bone regeneration (GBR) at the anterior region.

**Figure 1:**
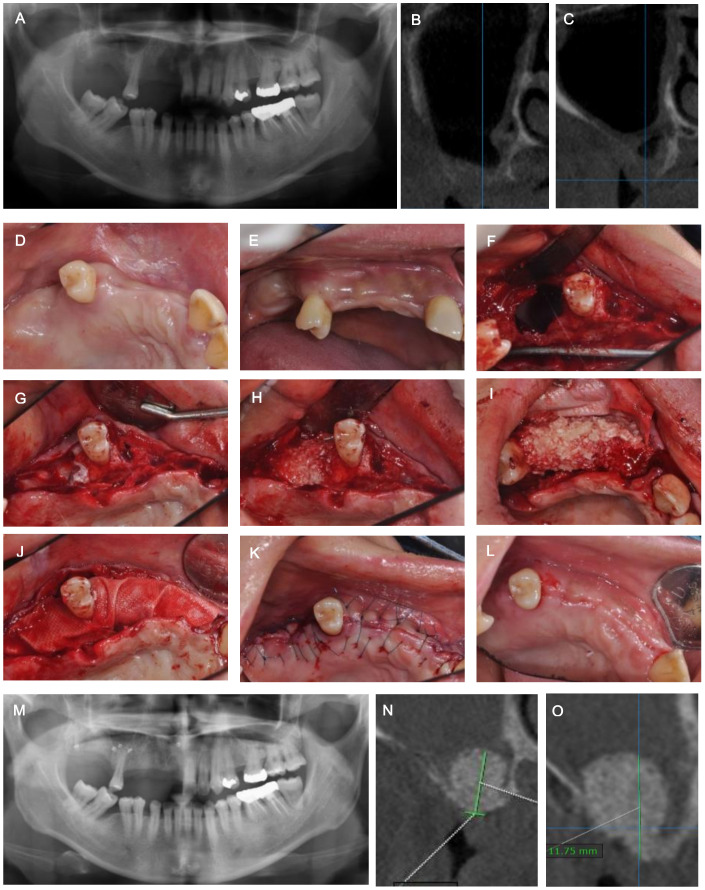
(**A**) Pre-operative panoramic view. (**B**) Pre-operative CT view of #17. (**C**) Pre-operative CT view of #16. (**D**,**E**) Pre-operative clinical view. (**F**) Oro-antral communication (OAC). (**G**) Collagen membrane application in sinus achieved by making pouch shape. (**H**) Sinus bone graft with allogenic bone material. (**I**) Horizontal ridge augmentation of #11-14 area. (**J**) Collagen membrane application on the crestal area. (**K**) Suturing. (**L**) Healing condition at 2 weeks postoperatively. (**M**) Panoramic view after OAC closure and bone augmentation. (**N**) Post-operative CT view of #17. (**O**) Post-operative CT view of #16.

First, the flap was elevated under the superior alveolar nerve block. The flap was then separated from the inflammatory tissue around the fistula and maxillary sinus membrane using a 15c blade. Maxillary sinus membrane perforation and an OAC (≥20 mm in diameter) were seen in the alveolar bone crest. After fixing the collagen membrane (Jason membrane^®^, Straumann, Zossen, Germany) to the external alveolar bone using three bone tacks, the barrier membrane was pushed into the maxillary sinus through the OAC area in the form of a pouch to close the OAC. Thereafter, the allograft (Do bone^®^, CGBio, Seongnam, Korea) was filled, and the alveolar crest area was covered with a collagen membrane (Ossix Plus^®^, Datum Dental Biotech, Lod, Israel). At the same time, a horizontal GBR was performed using the allograft (Do bone^®^, CGBio, Seongnam, Korea) and the collagen membrane (Ossix Plus^®^, Datum Dental Biotech, Lod, Israel) at the site of #11–14. After the periosteal releasing incision, primary wound closure was performed at the surgical site. The patient was prescribed pain relievers (Aceclofenac 100 mg, Dona-A ST, Seoul, Korea) and antibiotics (Augmentin 625 mg, Ilsung Pharm. Co., Seoul, Korea) twice and thrice a day, respectively, for a week, to prevent any infection at the site arising from systemic diseases and long-term steroid use. Two weeks post-surgery, clinical radiographic observation revealed a complete closure of the OAC and a successful outcome of the maxillary sinus bone graft (Figure 1).

**Figure 2:**
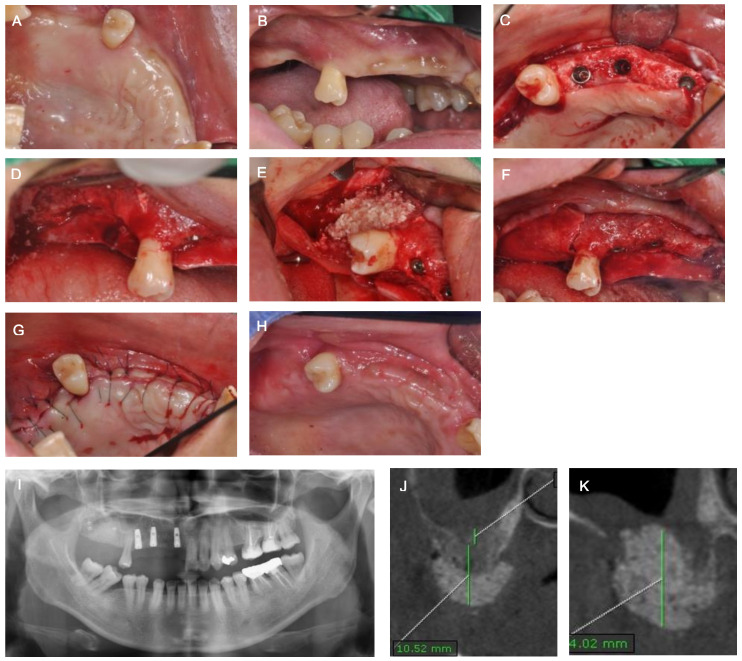
(**A**,**B**) Clinical view before bone augmentation and implantation. (**C**) Occlusal view after #11, 13 and 14 first-stage implant surgery (**D**) Buccal view after flap elevation at #16 and 17. (**E**) Bone graft at #16 and 17. (**F**) Absorbable membrane application at #16 and 17. (**G**) Suturing. (**H**) Healing condition at 2 weeks postoperatively. (**I**) Panoramic view after bone augmentation and implantation. (**J**) Post-operative CT view of #17. (**K**) Post-operative CT view of #16.

**Figure 3:**
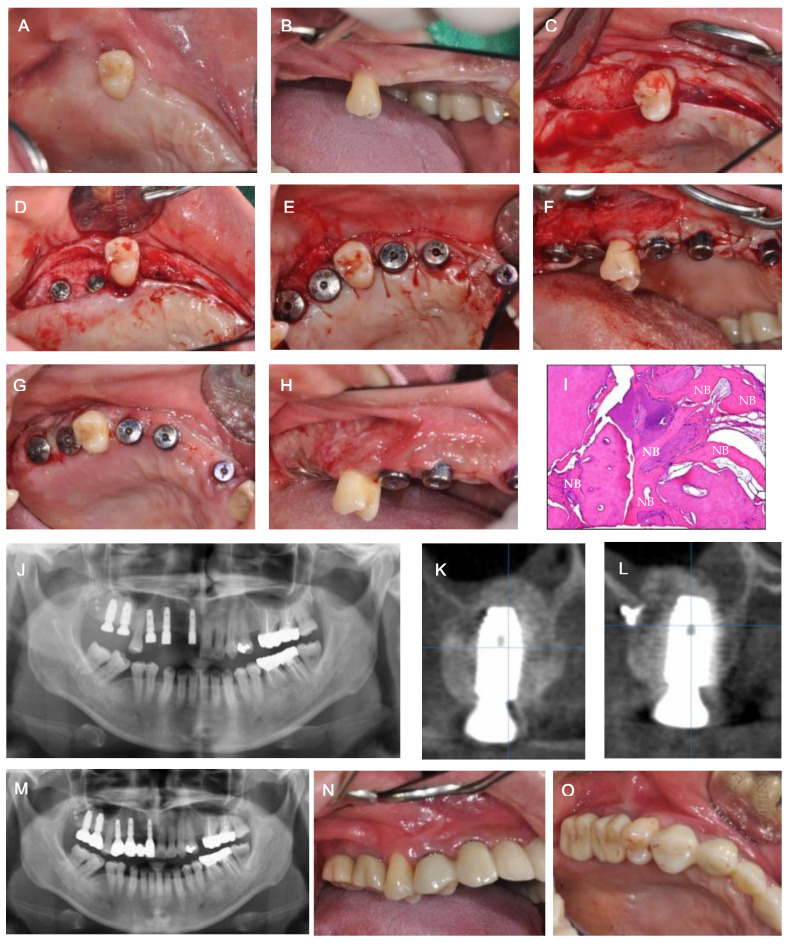
(**A**,**B**) Clinical view before first and second stages of implant surgery. (**C**) Occlusal view after flap elevation. (**D**) Occlusal view after #16 and 17 first stage implant surgery. (**E**) Occlusal view after implant second surgery. (**F**) Buccal view after performing modified periosteal fenestration. (**G**,**H**) Healing condition at 2 weeks post-operation. (**I**) Histological analysis. New bone formation (NB) was observed with no inflammatory tissue (Hematoxylin and eosin (H & E) stained; original magnification × 100). (**J**) Panoramic view after implantation. (**K**) Post-operative CT view of #17. (**L**) Post-operative CT view of #17. (**M**) Panoramic view after prosthesis. (**N**,**O**) Clinical view after prosthesis.

Six months post-operation, the first stage of implant surgery for #11, 13, and 14, and GBR for vertical ridge augmentation of #16 and 17, were performed simultaneously. The allograft (Do bone^®^, CGBio, Seongnam, Korea) was applied in the area of #16 and 17, which was covered with an absorbable membrane (Ossix Plus^®^, Datum Dental Biotech, Lod, Israel) and fixed with three bone tacks. Radiographically, the implant placement of #11, 13, and 14 and sufficient horizontal and vertical bone augmentation at #16 and 17 were observed following the implant surgery. After approximately 6 months from the implant surgery, the radiograph showed sufficient hard tissue volume in areas #16 and 17, and indicated the recovery of the maxillary sinus membrane (Figure 2). Thus, the second stage of implant surgery for #11, 13, and 14, and the first stage of implant surgery for #16 and 17 were executed. Previously, vestibular loss and a lack of buccal attached mucosa were observed with OAC closure and vertical GBR. Therefore, modified periosteal fenestration [11,12], which we first suggested as a free gingival graft alternative, was performed simultaneously with the implant surgery. The core biopsy was conducted before drilling at the site of #16 for implant placement. The biopsy was harvested through the alveolar at a depth of 10 mm using a trephine bur with an inner diameter of 2 mm. High primary stability was obtained when the implant was placed, and sufficient marginal bone width was confirmed in the buccal and lingual areas, as revealed through the radiographs. The harvested specimens were fixed using paraformaldehyde in 4% buffered saline, followed by demineralization. The specimens were then processed into paraffin blocks, and a microtome (RM2125RTS; Leica, Nussloch, Germany) was used for micro-sectioning. Next, hematoxylin and eosin staining was performed. The final prosthesis was restored after approximately 5 months. Following maxillary sinus elevation with OAC closure, no complications, such as maxillary sinusitis, were observed during the continuous management period of approximately 2 years. Histologic and histomorphometric analyses revealed the deposition of newly formed bone (NB) around the residual allogenic bone graft (RG), and satisfactory incorporation between the NB and RG. No special foreign body reactions or inflammatory signs were detected. The sample showed 27.3% NB, 29.4% RG, and 43.3% connective tissue (Figure 3).

## 3. Discussion

Several procedures describing the closure of OAC have been introduced, including the use of soft tissue flap, sinus elevation accompanied by bone grafting, and the use of platelet-rich fibrin [1,2,3,4]. In 2019, Parvini et al. [3] proposed that diverse factors, such as the presence of infection, the size of the defect, the timing of diagnosis, and the patient’s medical history should be considered to determine the surgical closure procedure. Surgical treatment is generally recommended for OAC or OAF; however, as the first choice, most surgeons follow the procedure that employs a soft tissue flap. The major disadvantages of using the buccal flap are the lowering of the oral vestibule and the insufficient attached mucosa. If the surgical site to be covered is wide, the possibility of failure is high. If the palatal flap is applied to the molar region, excessive tension is generated, and the resulting ischemia is likely to cause necrosis [5,6,7]. In particular, when OAC and OAF are closed with soft tissue, the most critical challenge for clinicians is the difficulty of the additional treatment of bony defects for implant placement, leading to a prolonged treatment period. In addition, adhesion between the oral mucosa and maxillary sinus membrane cannot be elevated without perforation of the maxillary sinus membrane [5,6].

Several methods have been proposed to compensate for the shortcomings of soft tissue closure. Proctor et al. [13] introduced OAF closure via an autogenous bone. Autogenous bones were collected from the extraction socket, mandibular symphysis, and iliac crest. The recovery of the maxillary bone defect and OAF closure through the autogenous bone showed successful results. However, this method has a limitation in that the residual alveolar bone must be of a sufficient height and width, and there must be a sound cortical bone layer, in order to obtain predictable results. Furthermore, additional donor site surgery is required, and the risk of recurrence is increased by the rapid resorption of the autogenous bone [13]. Isler et al. [14] reported the use of auricular cartilage, which offers the advantages of biocompatibility, high resistance to infection, ease of manipulation, and lack of absorption. Moreover, it has been proven that the fusion of such cartilage with the recipient site does not require vascularization, and hence, reduces the rate of transplant failure. It was reported that the auricular cartilage was a barrier to the oral mucosa and maxillary sinus mucosa, thereby enabling successful healing and the obtaining of predictable results [14]. Scattarella et al. [15] reported a method for recovering bone defects and OAF closure using allogeneic and heterogeneous bones by blocking the migration of the oral mucosa epithelium.

In the case of subsequent implant surgeries, a method for maxillary sinus elevation with OAF or OAC closure was introduced. Ogunsalu [14] reported a sandwich technique for OAC or OAF closure. This method involved constructing two absorbable membranes in an appropriate shape, followed by sewing three sides with absorbable silk to form a sandwich shape with one side open. Subsequently, the bone grafting was sandwiched between them and applied to the OAC area. This led to the successful closure of the OAC or OAF, and predictable bone formation was achieved after the implant’s placement [16].

**Figure 4:**
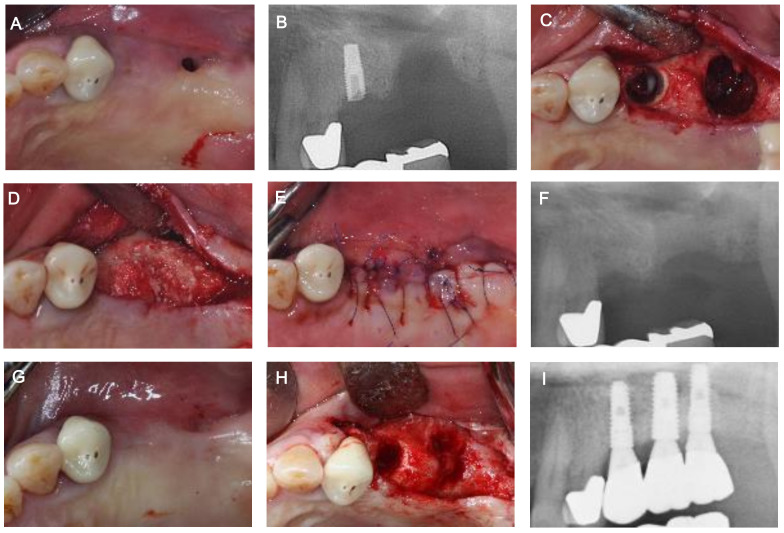
Another case of implant placement after oro-antral fistula (OAF) and oro-antral communication (OAC) closure using an absorbable collagen membrane in the form of a pouch. (**A**) Pre-operative clinical view of #27. (**B**) Pre-operative radiograph of #27. (**C**) Maxillary sinus membrane perforation and an OAC (≥10 mm in diameter). (**D**) Sinus bone graft with allogenic bone material after collagen membrane application in the sinus by making a pouch shape. (**E**) Suturing. (**F**) Post-operative radiograph of #27. (**G**) Healing condition at 2 weeks post-operatively. The OAF was treated. (**H**) Clinical view before first-stage implant surgery. Promising hard tissue development at the site of the OAC was observed. (**I**) Radiograph after prosthesis.

**Figure 5:**
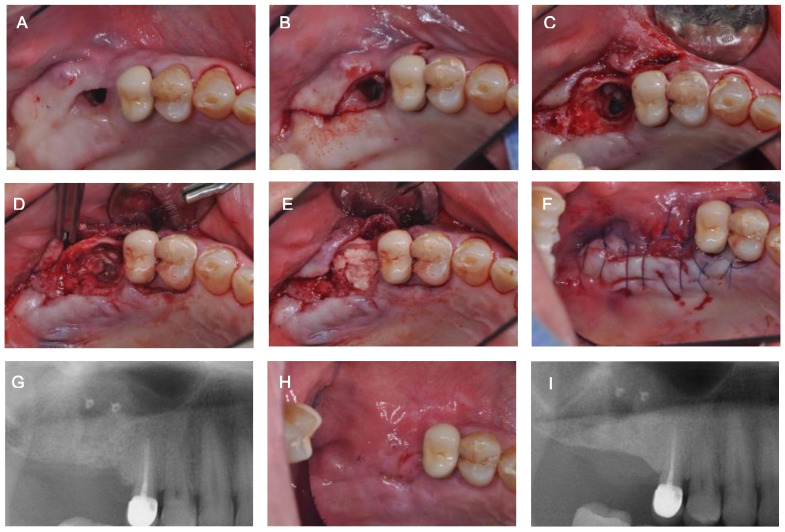
Another case of oro-antral fistula (OAF) and oro-antral communication (OAC) closure using an absorbable collagen membrane in the form of a pouch. (**A**) Pre-operative clinical view of #16. (**B**) Incision design for epithelial patency soft tissue removal. (**C**) Maxillary sinus membrane perforation and an OAC (≥6 mm in diameter). (**D**) Collagen membrane application in the sinus by making a pouch shape. (E) Sinus bone graft with allogenic bone material. (**F**) Suturing. (**G**) Post-operative radiograph of #16. (**H**) Healing condition at 2 weeks post-operatively. The OAF was treated. (**I**) Radiograph at 6 months post-operatively. Increased radiopacity was observed at the OAC site.

In the present study, a maxillary sinus bone graft was performed by applying an absorbable collagen membrane in the form of a pouch to the maxillary sinus through the OAC (Figure 4 and Figure 5). This was a modified method of the existing Loma Linda method [17,18], which was used for the treatment of a perforated maxillary sinus membrane during lateral approach maxillary sinus elevation and the sandwich method reported by Ogunsalu [16]. In the present study, maxillary sinus perforation of more than 20 mm was observed, but the results were predictable in the recovery of the maxillary sinus mucosa and OAC closure. Compared to the existing methods, our method has the following advantages. Unlike the sandwich method [16], it was possible to easily perform both maxillary sinus perforation closure and maxillary sinus bone grafting by applying an absorbable membrane fixed with bone tacks inside of the maxillary sinus through the opening in the form of a pouch. Additionally, it was possible to create an environment more favorable for the progression of surgical procedures, including GBR and the placement of implants in the same area.

## 4. Conclusions

As per our present study, for an OAC of 20 mm or more, an effective treatment method has been introduced. The method entails the application of a collagen barrier membrane in the form of a pouch to the OAC area using bone tacks and simultaneous maxillary sinus bone grafting followed by implant placement. However, this procedure necessitated thorough inflammation control, since it was accompanied by bone grafting. In the case of existing maxillary sinusitis before surgery, or the occurrence of any exudation, prior control of inflammation is mandatory before proceeding to treatment. Long-term observation may be needed, and long-term studies involving bone grafting, along with OAC closure, are warranted in many cases.
